# Thalamic Subregion Alterations and Short-Chain Fatty Acids in Schizophrenia and Ultra-High-Risk Individuals: A Cross-Sectional Study

**DOI:** 10.31083/AP49150

**Published:** 2026-06-29

**Authors:** Huiqing Peng, Xiaogang Chen, Ying He, Shuting Chen, Zongchang Li, Chunwang Li, Ke Jin, Liu Yuan, Xiaoqian Ma

**Affiliations:** ^1^Department of Psychiatry, National Clinical Research Center for Mental Disorders, and National Center for Mental Disorders, The Second Xiangya Hospital of Central South University, 410011 Changsha, Hunan, China; ^2^Hunan Key Laboratory of Psychiatry and Mental Health, 410011 Changsha, Hunan, China; ^3^Department of Psychiatry, Second Affiliated Hospital of Zhejiang University, 310009 Hangzhou, Zhejiang, China; ^4^Department of Radiology, Hunan Children’s Hospital, 410007 Changsha, Hunan, China

**Keywords:** clinical high risk, magnetic resonance imaging, schizophrenia, short-chain fatty acids, thalamus

## Abstract

**Background:**

Thalamic abnormalities have been implicated in schizophrenia, but their early role remains unclear. This study investigated structural and functional alterations of thalamic subregions in drug-naïve first-episode schizophrenia (FES) patients and ultra-high-risk (UHR) individuals, and explored their associations with serum short-chain fatty acids (SCFAs).

**Methods:**

This cross-sectional study included 102 FES patients, 72 UHR individuals, and 69 healthy controls (HCs). Structural magnetic resonance imaging (MRI) data were available for all participants, and functional MRI and SCFA measurements were conducted in subgroups (functional MRI (fMRI): 76 FES, 63 UHR, 61 HC; SCFA: 59 FES, 51 UHR, 40 HC).

**Results:**

Thalamic volume was smaller in FES compared to HCs, with atrophy present specifically at the psychotic stage, particularly affecting the right thalamus and nuclei including the mediodorsal medial magnocellular (MDm), ventromedial (VM), and ventral posterolateral (VPL). Functional connectivity (FC) disruptions were observed in the left sensory thalamus with cortico-striatal-thalamic circuits and in the right occipital thalamus with fronto-temporal regions. Thalamic volumetric deficits correlated with negative and disorganized symptoms in both clinical groups. Serum SCFAs showed several significant associations: in the UHR group, FC between the right occipital thalamus and the left medial frontal cortex was negatively associated with acetic acid and total SCFA levels; in FES patients, centromedian nucleus volume was positively correlated with acetic acid; and in HCs, butyric acid was inversely correlated with the volume of the mediodorsal (MD) lateral nucleus.

**Conclusions:**

These findings highlight early thalamic subregional alterations in psychosis risk and co-occurring changes in gut-derived metabolites. Whether and how these changes are related remains a question for future research.

## Main Points

1. Progressive thalamic atrophy from healthy controls (HCs) to first-episode schizophrenia (FES) patients, especially in right-sided nuclei.

2. Thalamic subregions show distinct structural and functional alterations in both the ultra-high-risk (UHR) and FES groups.

3. Volume loss correlates with symptom severity in negative and disorganized domains.

4. Short-chain fatty acids (SCFAs) are associated with thalamic structure and function, hinting at gut–brain interactions in early psychosis.

## 1. Introduction

Schizophrenia (SCZ) is a severe neurodevelopmental disorder typically emerging in adolescence or early adulthood, characterized by psychiatric symptoms and cognitive deficits [1,2]. Its etiology involves a combination of genetic predisposition and environmental risk, leading to a range of brain structural abnormalities. Gray matter deficits and functional disruptions are widely observed in SCZ [3], including abnormalities in subcortical structures such as the hippocampus, thalamus, and caudate. Studies have shown that these abnormalities may even precede the onset of clinical symptoms [4,5]. That fact suggested that structural alterations in subcortical nuclei may be involved in the pathophysiological mechanisms of SCZ [6,7,8]. Given the central role of subcortical structures in early disease development and their potential as therapeutic targets, there is a pressing need to conduct well-controlled neuroimaging studies in carefully characterized samples to further elucidate the contribution of subcortical structures to disease mechanisms and to identify novel intervention opportunities.

Although numerous studies have investigated thalamic alterations in SCZ, their findings remain highly inconsistent, likely due to substantial heterogeneity across study samples. For example, Wang et al. [9] reported increased functional connectivity (FC) in first-episode schizophrenia (FES) patients, a finding that contrasts with studies using mixed-stage SCZ samples [9]. Additionally, thalamic volume changes are primarily left-sided in SCZ, but more pronounced on the right side in FES. These inconsistent findings hindered our understanding of disease mechanisms, thus requiring validation and clarification. Drug-naïve FES patients offer a clearer picture, because there are no confounding effects of disease duration and medication, thereby offering a more accurate exploration of the brain structural and functional changes directly attributed to the disease.

In addition to the post-onset stage, the prodromal phase of SCZ, often referred to as the ultra-high risk (UHR) stage, also provides valuable insights into the underlying mechanisms of the disorder. Studies have shown that UHR individuals exhibited lower volumes and altered FC, particularly in the striatum and thalamus, during the early stages of psychosis [4]. These findings aligned with the view that the UHR stage represents a critical period in which early neurodevelopmental vulnerabilities may become functionally manifested, setting the stage for potential illness progression. The cerebello-thalamo-cortical pathway has been suggested as a location for a trait-specific biomarker for SCZ [10]. Fryer et al. [5] showed that thalamic dysconnectivity occurred before the onset of illness, and that these changes were more pronounced during the early stages. Furthermore, structural deficits in hippocampus, amygdala and thalamus have been observed in UHR individuals [11,12]. These results suggested that structural and functional abnormalities in subcortical structures, particularly in thalamus, may already be present at the UHR stage [13,14,15,16]. In addition, studies found that reduced thalamic volume, disrupted thalamus-cortical dysconnectivity, and aberrant laterality also consistently occurred in FES patients [15,17,18,19,20,21,22]. Therefore, thalamic abnormalities may span the entire course of SCZ, both pre- and post-onset. Notably, the thalamus is a highly heterogeneous structure, composed of multiple nuclei with distinct cellular compositions and specific anatomical connections to both the cortex and subcortex. Several studies have suggested that abnormalities in the anteroventral, centro-median, medial dorsal, and pulvinar nuclei are central to the pathology hypothesis of SCZ [23,24,25,26,27]. However, many SCZ studies considered the thalamus as a whole and overlooked its heterogeneous structure, especially in UHR and FES studies. Therefore, further research is needed.

Recent evidence has suggested that gut microbiota-derived short-chain fatty acids (SCFAs) may play a role in the pathophysiology of SCZ through systemic immunomodulatory and epigenetic mechanisms [28,29,30,31]. Our previous work further indicated that specific anti-inflammatory SCFAs were lower than normal in both UHR and FES individuals [32]. Critically, as a central hub for sensory integration and corticocortical communication, the thalamus, particularly its anterior and mediodorsal nuclei connected to the prefrontal and limbic systems, is a site of early neurodevelopmental disruption in psychosis [19]. These circuits are known to be sensitive to metabolic and neuroinflammatory states, which SCFAs are positioned to modulate. However, direct evidence linking SCFAs to thalamic integrity in SCZ is lacking.

To address this gap, we conducted a multimodal study in both UHR and FES individuals. We hypothesized that: (1) Structural and functional abnormalities in the thalamus occur at an early stage of SCZ, even prior to the onset of SCZ, contributing to the subclinical or clinical symptoms in UHR and FES individuals; (2) SCFAs relate to SCZ pathophysiology not in a diffuse manner, but specifically through associations with the integrity of discrete thalamic subregions and their corticothalamic circuits. In the present study, we explored thalamic nuclei alterations at early stages of SCZ and measured their neuroimmune mechanisms.

## 2. Materials and Methods

### 2.1 Participants

Patients with FES and individuals at UHR of SCZ were recruited from the outpatient units of the Department of Psychiatry, Second Xiangya Hospital of Central South University (Changsha, China) between 2015 and 2022. Participants were aged 13–30 years. FES patients were diagnosed using the structured clinical interview (SCID) according to Diagnostic and Statistical Manual of Mental Disorders, Fourth Edition (DSM-IV) criteria for SCZ. All included FES patients met the criteria for first-episode SCZ, had a duration of illness of less than two years (average illness duration: 8.27 ± 3.21 months), and had never received any psychotropic medication prior to assessment.

UHR individuals were evaluated using the structured interview for prodromal syndromes (SIPS) [33], and met at least one prodromal syndrome: Attenuated Positive Syndrome (APS), Brief Intermittent Psychotic Syndrome (BIPS) and Genetic Risk and Deterioration Syndrome (GRDS).

Clinical symptoms were assessed using the Positive and Negative Syndrome Scale (PANSS) for FES patients and the Scale of Prodromal Symptoms (SOPS) for UHR individuals.

HCs were recruited from local schools in Changsha through advertisements. All HC participants were screened by two psychiatrists using the Structured Clinical Interview for DSM-IV, Non-Patient Version (SCID-NP), and none met criteria for any current or lifetime psychiatric disorder.

The exclusion criteria for all participants were as follows: (1) history of antipsychotic treatment; (2) any contraindications to MRI scanning; (3) substance abuse or dependence disorder; (4) history of loss of consciousness for >5 min; (5) current or chronic neurological disorder; (6) comorbidity with other DSM-IV psychiatric diagnoses.

For the SCFA analysis, data were available for a subset of participants from the same MRI cohort. All participants included in the SCFA analysis met the same inclusion and exclusion criteria as the MRI sample, with additional exclusions for factors known to affect SCFA levels, including gastrointestinal or endocrine diseases, serious organ disorders, and use of alcohol, antibiotics, probiotics, or any other medications within the last 3 months.

Each participant completed MRI scanning, blood sampling, and clinical assessments within 24 h of enrollment. Written informed consent was obtained from all adults and from parents/guardians of minors, with adolescent assent. This study was approved by Ethics Committee of the Second Xiangya Hospital, Central South University (Ethics Committee No. 2021YFE0191400) and carried out in accordance with the Declaration of Helsinki.

### 2.2 MRI Data Acquisition

The MR scans were conducted using a 3.0T Magnetic Resonance (MR) machine (Siemens, Skyra, Erlangen, Germany) equipped with a 16-channel array coil at the Magnetic Imaging Centre of Hunan Children’s Hospital, Changsha, Hunan, China. All participants were instructed to remain awake with their eyes closed during the scanning. The functional imaging sequence parameters were as follows: repetition time (TR) = 2000 ms; echo time (TE) = 30 ms; flip angle = 90°; slice number = 36; slice thickness = 3.4 mm; voxel size = 3.4 × 3.4 × 3.4 mm^3^; field of view (FOV) = 256 × 256 mm. The slice acquisition order was interleaved, and the phase-encoding direction was anterior-to-posterior. Each functional run contained 250 image volumes, resulting in a functional scanning time of 500 s. No field maps or distortion correction were applied. For structural imaging, high-resolution T1-weighted images were acquired with the following parameters: TR = 2530 ms; TE = 2.33 ms; flip angle = 7°; slice number = 192; slice thickness = 1 mm; voxel size = 1 × 1 × 1 mm^3^; FOV = 256 × 256 mm. Structural images were acquired with axial slice orientation, and no distortion correction was applied.

### 2.3 Structural MRI (sMRI) Data Processing

Structural T1-weighted images were processed using Freesurfer (7.2.0, Massachusetts General Hospital, Charlestown, MA, USA, http://surfer.nmr.mgh.harvard.edu/) with the standard recon-all pipeline [34]. All reconstructed outputs were visually inspected to ensure segmentation accuracy. Subcortical segmentation and estimated total intracranial volume (eTIV) were obtained using the *aseg* and *asegstats* modules. Thalamic nuclei were segmented using the FreeSurfer *segmentThalamicNuclei* command, which is based on the probabilistic histology-derived thalamic nuclei atlas by Iglesias et al. [35], yielding 25 nuclei per hemisphere. Quality control was performed for each subject by two independent raters who were blind to group information. The following aspects were evaluated: skull stripping accuracy, intensity normalization, white matter mask integrity, subcortical boundary alignment, and thalamic nuclei label placement. Scans were excluded if they showed: (1) uncorrectable skull-stripping errors; (2) severe white matter or subcortical spanning >4 consecutive slices; or (3) major mislabeling of thalamic nuclei that could not be corrected. Disagreements were resolved by consensus. Only scans passing all quality control criteria were included in the final analyses.

### 2.4 Function MRI (fMRI) Data Processing

The functional MRI (fMRI) data processing used the Data Processing Assistant for Resting-state fMRI [36] (DPARSF 4.1, State Key Laboratory of Cognitive Neuroscience and Learning, Beijing Normal University, Beijing, China, http://www.restfmri.net). The procedures were as follows: (1) remove the first 10 time series of each individual to get a stabilized scan; (2) slice timing; (3) realign to the middle volume; (4) normalize the image to Montreal Neurological Institute (MNI) coordinates space (DARTEL normalization parameters); (5) resample to 3 × 3 × 3 mm^3^. (6) spatial smoothing conducted by a 6-mm full-width at half-maximum (FWHM) Gaussian kernel; (7) linear-drift correction and nuisance-covariates regression (includes cerebrospinal fluid and white matter signals applied to minimize confounder effects); (8) adopt Friston’s 24-parameter model to eliminate head motion. Participants were excluded if their maximum head motion exceeded 2 mm in translation or 2° in rotation during the scan. Framewise displacement (FD) was calculated to represent the head motion at each time point; signals from each “bad” time point (defined as FD >0.5 mm), along with the signals 1 frame before and 2 frames after the bad time point, were added as separate regressors for regression; (9) the fMRI data passed a temporal filter (0.01–0.08Hz) to eliminated low-frequency drift and high frequency physiological noise. The human Brainnetome Atlas [37] was used to define regions of interest (ROI), dividing the thalamus into 16 subregions per hemisphere. For each participant, the mean time series was extracted from each ROI, and Pearson’s correlation coefficients were computed between the representative time series and the time series of all other voxels in the whole brain. Correlation coefficients were transformed to z values using Fisher’s r-to-z transformation for statistical analyses.

### 2.5 Blood Sample Collection and SCFAs Data Extraction

Blood samples (6 mL) were voluntarily provided by a subset of participants. Participants were required to fast at least 8 h prior to collection, and to avoid coffee, alcohol and strenuous exercise for at least 30 min before the morning blood draw. After blood centrifugation (3000 r/min, 10 min), the serum was stored at –80 ℃ for further processing. The details of the serum SCFA data extraction process are presented in our previously published study [32].

### 2.6 Statistical Analysis

Statistical analyses were performed using SPSS 27.0 (IBM Corp., Armonk, NY, USA) and MATLAB R2022b (MathWorks, Natick, MA, USA). Descriptive analyses summarized demographic and clinical data across the FES, UHR, and HC groups. The Chi-square test was used for sex. Age, years of education, PANSS scores, and SOPS scores were analyzed using one-way ANOVA or t-tests, based on data distribution and described as mean ± standard deviation. PANSS scores were decomposed using the five-factor model [38], including positive symptoms, negative symptoms, disorganization, depression/anxiety, and excitability/hostility. Serum SCFA data were processed using the same procedures described in our previous study [32].

Hemispheric asymmetry was quantified using the laterality index (LI): LI = (left − right) / (left + right). Both LI and its absolute value (|LI|) were analyzed.

Imaging measures, including whole thalamus volumes, thalamic nuclei volumes (25 nuclei), functional connectivity, and laterality indices, were analyzed using general linear models (GLMs). Age, sex, and years of education were entered simultaneously as covariates in all models, with eTIV additionally included for volumetric measures, interactions with group are tested in **Supplementary Table 1**. First, a global group effect was tested for each model, and the *p*-values of the global tests were corrected using false discovery rate (FDR) correction, with statistical significance set at *p*-_FDR_ < 0.05, effect sizes were reported as *η*^2^. Second, for measures that survived FDR correction, post-hoc pairwise comparisons (FES vs. UHR, FES vs. HC, UHR vs. HC) were performed using Bonferroni correction, the adjusted *p*-value (*p*-_Bonf_) was calculated by multiplying the raw *p*-value by 3 (*p*-_Bonf_ = 3*p-raw), and comparisons with *p*-_Bonf_ < 0.05 were considered statistically significant.

Imaging measures showing significant group differences were further examined using partial correlation analysis with clinical scores, and SCFA levels within each group, as an exploratory analysis. FDR correction was applied across all correlations tested within each domain, with *p*-_FDR_ < 0.05 considered significant. Age, sex, years of education, and eTIV (for volumetric measures) were simultaneously included as covariates.

## 3. Results

### 3.1 Demographic and Clinical Characteristics

**Fig. 1.**
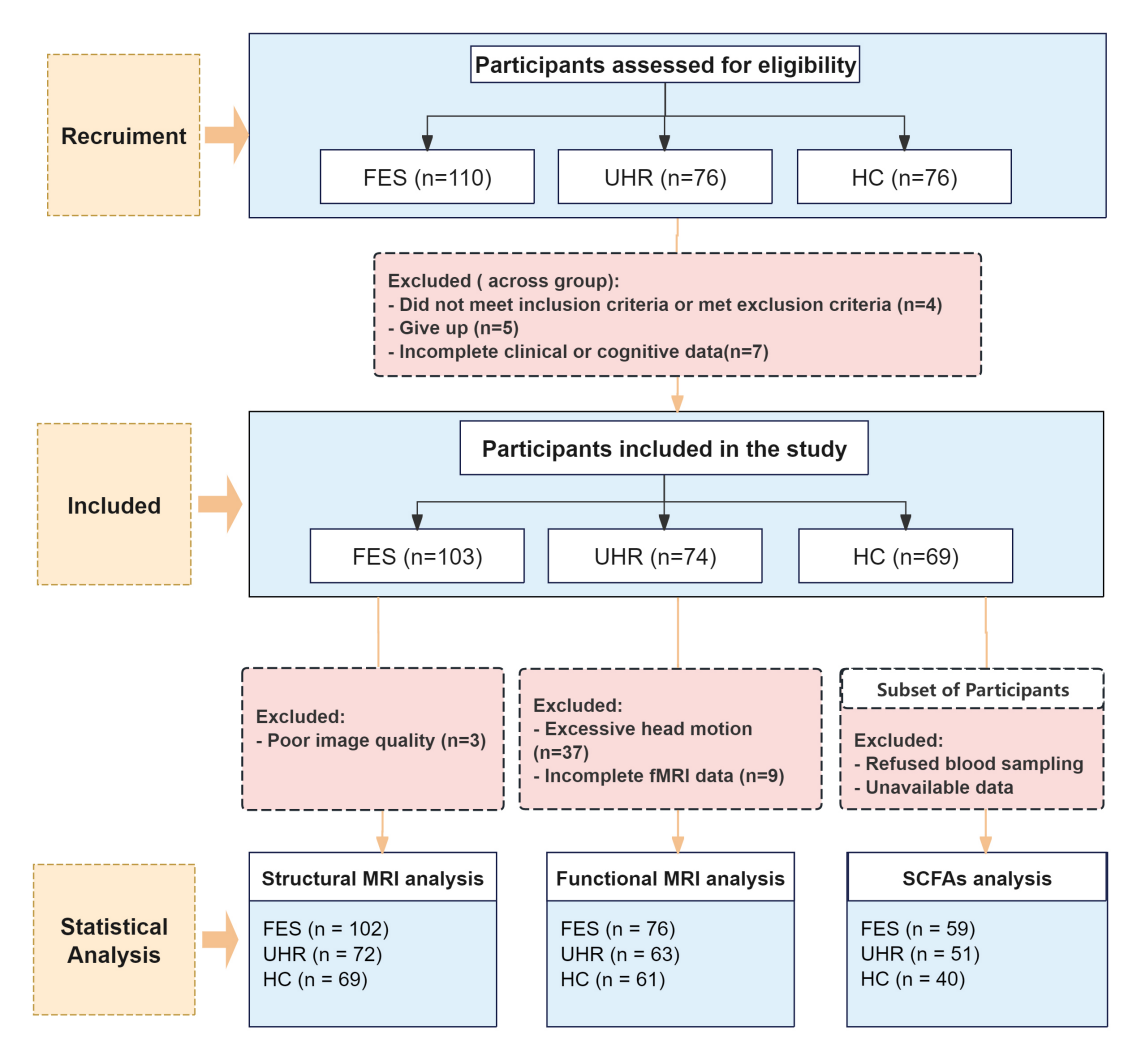
**Flowchart of participant recruitment, exclusion, and inclusion in the study**. Note: Flowchart illustrating participant recruitment, eligibility assessment, and derivation of analytic samples for the first-episode schizophrenia (FES), ultra-high risk (UHR), and healthy control (HC) groups. Exclusions were applied according to predefined eligibility and data-quality criteria. Final sample sizes included in structural MRI, functional MRI, and short-chain fatty acid (SCFA) analyses are shown.

A total of 243 participants were enrolled, including 102 FES patients, 72 UHR subjects, and 69 sex- matched HCs. The participant selection process is summarized in Fig. 1. Demographic and clinical characteristics of participants of all groups are described in Table 1. Demographic and clinical characteristics of the participant subsets analyzed for fMRI, sMRI, and SCFAs are compared in **Supplementary Tables 2.1–2.3**. When comparing the FES, UHR and HC groups, no significant differences were found in sex (χ^2^ = 0.23, *p* = 0.890). However, significant differences were noted in age (*F *= 6.21, *p *< 0.05) and years of education (*F *= 16.14, *p *< 0.05).

**Table 1.** **Demographic and clinical characteristics of all subjects**.

		FES (*n *= 102)	UHR (*n *= 72)	HC (*n *= 69)	F/χ^2^	*p-*value	Post-hoc (Bonferroni-correction)
Age		21.23 ± 5.54	19.00 ± 4.72	21.78 ± 4.62	6.21	0.002^a^	FES > UHR, *p*-_Bonf_ = 0.014; HC > UHR, *p*-_Bonf_ = 0.004
Sex (male/female)		59/43	39/33	39/30	0.23	0.890^b^	
The years of education		11.75 ± 2.84	11.06 ± 2.78	13.58 ± 2.53	16.14	<0.001^a^	HC > FES, *p*-_Bonf_ < 0.001; HC > UHR, *p*-_Bonf_ < 0.001
*PANSS*							
	Positive symptom	21.88 ± 6.85	-	-	-	-	
	Negative symptom	21.48 ± 6.32	-	-	-	-	
	Disorganization	27.08 ± 7.19	-	-	-	-	
	Depression/Anxiety	18.92 ± 5.36	-	-	-	-	
	Excitability/Hostility	22.10 ± 5.51	-	-	-	-	
*SOPS*							
	SOPS-P score	-	10.78 ± 4.93	-	-	-	
	SOPS-N score	-	12.11 ± 5.16	-	-	-	
	SOPS-D score	-	5.08 ± 2.67	-	-	-	
	SOPS-G score	-	4.63 ± 2.94	-	-	-	

Note: PANSS, Positive and Negative Syndrome Scale; SOPS, scale of prodromal symptoms; P, positive symptom; N, negative symptom; D, disorganized symptom; G, general symptom. The data are described as (Mean ± SD). Post-hoc analyses were corrected by Bonferroni. ^a^: The* p-*value was obtained by a one-way analysis of variance (ANOVA). ^b^: The *p-*values were obtained by the chi-square test.

The group difference of SCFA levels among FES, UHR and HC are described in Table 2. A significant difference was found in the years of education (*F *= 7.19, *p *< 0.05), but no significant differences were observed in sex or age. Moreover, valeric acid (*F *= 6.35, *p*-_Bonf_ = 0.042), and caproic acid (*F *= 8.17, *p*-_Bonf_ = 0.017), showed significant differences among groups.

**Table 2.** **Short-chain fatty acids (SCFAs) among FES, UHR and HC**.

	FES	UHR	HC	H/F	*p*-_Bonf_	dCohen	Post-hoc
Acetic acid	1.93 (1.38, 2.91)	1.87 (1.38, 2.72)	2.14 ± 1.08	0.31	0.852		
Butyric acid	0.05 (0.03, 0.08)	0.05 (0.03, 0.08)	0.07 ± 0.03	3.82	0.148		
Isovaleric acid	0.04 (0.00, 0.05)	0.04 (0.02, 0.06)	0.04 (0.03, 0.06)	3.20	0.202		
Valeric acid	0.00 (0.00, 0.06)	0.03 (0.00, 0.07)	0.04 ± 0.03	6.35	0.042	0.349	HC > FES, *p*-_Bonf_ = 0.036
Caproic acid	0.05 (0.00, 0.06)	0.03 (0.00, 0.05)	0.05 (0.04, 0.08)	8.17	0.017	0.422	HC > UHR, *p*-_Bonf_ = 0.016
Total SCFAs	2.39 ± 1.14	2.23 ± 1.00	2.32 ± 1.09	0.26	0.769		

Note: Data are presented as mean ± SD for normally distributed variables and median (25th, 75th percentiles) for non-normally distributed variables. Acetic acid, butyric acid, isovaleric acid, valeric acid, and caproic acid were tested by the Kruskal-Wallis (H statistic). One-way ANOVA was used for total SCFAs (F statistic). Bonferroni correction was applied, and *p*-_Bonf_ < 0.05 was considered statistically significant.

### 3.2 Group Differences in Thalamus and Thalamic Nuclei Volumes

#### 3.2.1 Thalamus volume

**Fig. 2.**
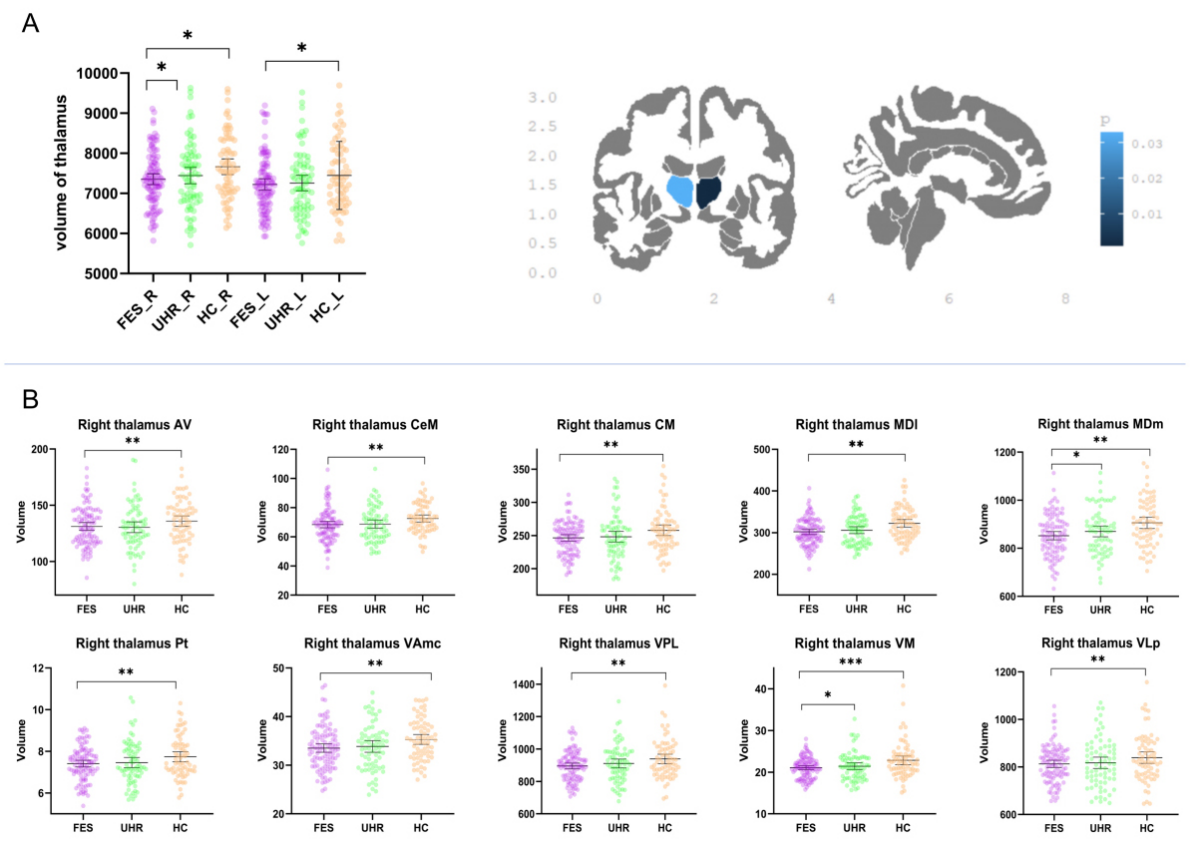
**Significant group differences in the thalamus and thalamic nuclei volumes**. Note: FES = 102, UHR = 72, HC = 69. (A) Left and right thalamic volumes in FES, UHR, and HC groups. (B) Significant thalamic nuclei volume differences among FES, UHR, and HC groups. The horizontal line and bars represent the mean and the 95% confidence interval. *: *p*-_Bonf_ < 0.05, **: *p*-_Bonf_ < 0.01, ***: *p*-_Bonf_ < 0.001 (Bonferroni-adjusted). Abbreviations are provided in **Supplementary Table 3**.

A significant group effect was observed for the left (*F* = 3.47, *p*-_FDR_ = 0.033) and right thalamus volumes (*F* = 7.03, *p*-_FDR _= 0.002). Post-hoc pairwise comparisons indicated that FES patients had smaller bilateral thalamic volume than did HCs (left: *p*-_Bonf_ = 0.045, right: *p*-_Bonf_ = 0.001), and smaller right thalamus volume than did UHR (*p*-_Bonf_ = 0.043). No significant differences were observed between the UHR and HC groups (Fig. 2, Table 3).

**Table 3.** **Significant group differences in thalamus and nuclei volumes**.

Hemisphere/Group	Region	Linear model (FES vs UHR vs HC)			Post-hoc (Bonferroni-adjusted)	95% CI
		F	*p*-_FDR_	*η*^2^		[Lower, Upper]
Left	thalamus	3.47	0.033*	0.029	FES<HC, *p*-_Bonf_ = 0.045	[–485.11, –3.85]
Right	thalamus	7.03	0.002**	0.056	FES<HC, *p*-_Bonf_ = 0.001; FES<UHR, *p*-_Bonf_ = 0.043	[–564.89, –106.73] [–454.64, –5.57]
Right Thalamic nuclei						
Anterior	AV	4.527	0.033*	0.037	FES<HC, *p*-_Bonf_ = 0.009	[–13.53, –1.44]
Intralaminar	CeM	4.478	0.033*	0.037	FES<HC, *p*-_Bonf_ = 0.009	[–8.79, –0.94]
Intralaminar	CM	5.758	0.016*	0.047	FES<HC, *p*-_Bonf_ = 0.005	[–21.22, –2.98]
Medial	MDl	8.907	0.002**	0.070	FES<HC, *p*-_Bonf_ < 0.001	[–32.19, –8.75]
Medial	MDm	9.406	0.002**	0.074	FES<HC, *p*-_Bonf_ < 0.001; FES<UHR, *p*-_Bonf_ = 0.041	[–83.77, –22.87]; [–60.56, –0.87]
Medial	Pt	5.461	0.018*	0.044	FES<HC, *p*-_Bonf_ = 0.005	[–0.67, –0.09]
Ventral	VAmc	6.343	0.010*	0.051	FES<HC, *p*-_Bonf_ = 0.002	[–3.39, –0.63]
Ventral	VLp	4.184	0.040*	0.034	FES<HC, *p*-_Bonf_ = 0.03	[–55.75, –2.01]
Ventral	VM	8.976	0.002**	0.071	FES<HC, *p*-_Bonf_ < 0.001; FES<UHR, *p*-_Bonf_ = 0.035	[–2.89, –0.75]; [–2.16, –0.06]
Ventral	VPL	6.903	0.006**	0.055	FES<HC, *p*-_Bonf_ = 0.005; FES<UHR, *p*-_Bonf_ = 0.01	[–76.47, –10.33] [–72.48, –7.64]
|Laterality Index|						
	VM	6.176	0.002	0.022	FES<HC, *p*-_Bonf_ = 0.002	[–0.04, –0.01]

Note: Group-level significance was determined using false discovery rate (FDR) correction: * *p*-_FDR_ < 0.05, ** *p*-_FDR_ < 0.01. Post-hoc pairwise *p*-_Bonf_ values were adjusted by Bonferroni correction: *p*-_Bonf_ < 0.05 was considered significant. Abbreviations are provided in **Supplementary Table 3**.

#### 3.2.2 Nuclei volume

At the nuclei level, 10 of 25 right thalamic nuclei showed a significant global group effect after FDR correction across all right-sided nuclei (all *p*-_FDR_ < 0.05). These included AV (*F* = 4.53, *p*-_FDR_ = 0.033), CeM (*F* = 4.48, *p*-_FDR_ = 0.033), CM (*F* = 5.76, *p*-_FDR_ = 0.016), MDl (*F* = 8.91, *p*-_FDR_ = 0.002), MDm (*F* = 9.41, *p*-_FDR_ = 0.002), Pt (*F* = 5.46, *p*-_FDR_ = 0.018), VAmc (*F* = 6.34, *p*-_FDR_ = 0.010), VLp (*F* = 4.18, *p*-_FDR_ = 0.040), VM (*F* = 8.98, *p*-_FDR_ = 0.002), and VPL (*F* = 6.90, *p*-_FDR_ = 0.006). Post-hoc comparisons were conducted for these significant regions, FES patients had smaller volumes than HC in all 10 nuclei (all *p*-_Bonf_ < 0.05): AV (*p*-_Bonf_ = 0.009), CeM (*p*-_Bonf_ = 0.009), CM (*p*-_Bonf_ = 0.005), MDl (*p*-_Bonf_ < 0.001), MDm (*p*-_Bonf_ < 0.001), Pt (*p*-_Bonf_ = 0.005), VAmc (*p*-_Bonf_ = 0.002), VLp (*p*-_Bonf_ = 0.03), VM (*p*-_Bonf_ < 0.001), and VPL (*p*-_Bonf_ = 0.005). Compared with UHR group, FES patients showed smaller volumes in the MDm (*p*-_Bonf_ = 0.041), VM (*p*-_Bonf_ = 0.035) and VPL (*p*-_Bonf_ = 0.01). No significant differences were found for left thalamic nuclei (Fig. 2, Table 3).

### 3.3 Functional Connectivity of Thalamic Subregions

#### 3.3.1 ROI5, Left Sensory Thalamus

**Fig. 3.**
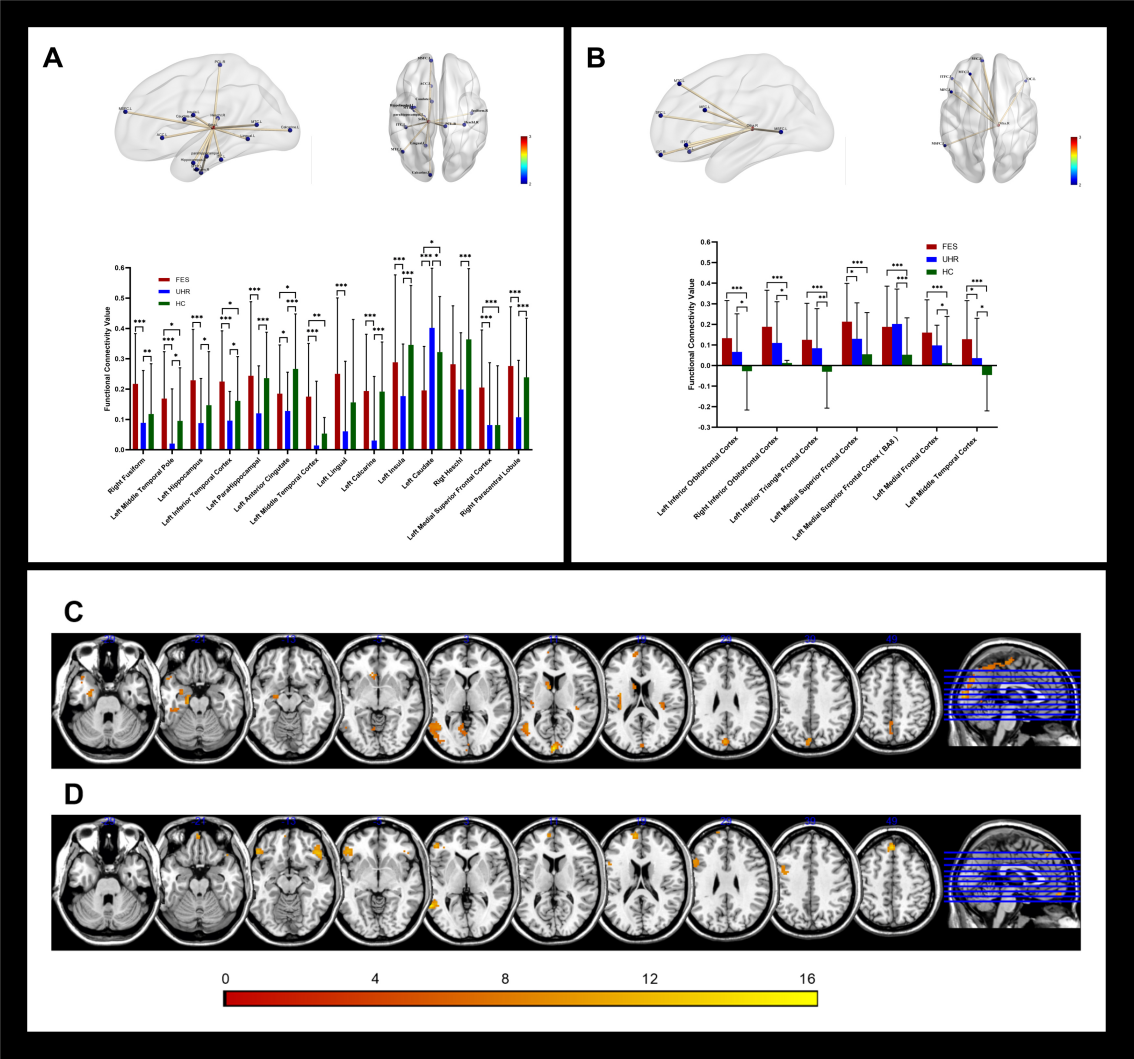
**Significant seed-to-region functional connectivity in FES, UHR, and HC groups**. Note: FES = 76, UHR = 63, HC = 61. (A,C) Functional connectivity using regions of interest (ROI) 5 as the seed ROI. (B,D) Functional connectivity using ROI 12 as the seed ROI. *: *p*-_Bonf_ < 0.05, **: *p*-_Bonf_ < 0.01, ***: *p*-_Bonf_ < 0.001 (Bonferroni-adjusted). FES, drug-naive first episode of schizophrenia; UHR, ultra-high risk; HC, healthy control. Abbreviations are provided in **Supplementary Table 3**.

When the left sensory thalamus (Stha. L, ROI5) was used as the seed ROI, a significant main effect of group was observed for FC with multiple cortical and subcortical regions after FDR correction (Fig. 3, **Supplementary Table 4**). Post-hoc analyses, adjusted using Bonferroni correction, showed that FES patients exhibited significantly greater FC between Stha. L and several regions, including the right fusiform gyrus, left middle temporal pole, left hippocampus, left inferior temporal cortex, left parahippocampal gyrus, left middle temporal cortex, left lingual gyrus, left calcarine cortex, left insula, right Heschl’s gyrus, left medial superior frontal cortex, and right paracentral lobule, than did either the UHR or the HC group (**Supplementary Table 4**). In contrast, the UHR group showed significantly lower FC in these regions than did both the FES and HC groups. Additionally, FC between Stha. L and the left caudate nucleus were significantly higher in the UHR group than in both the FES and HC groups. FC between Stha. L and the anterior cingulate cortex (ACC) were significantly lower in FES patients and further weakened in UHR individuals, with the strongest connectivity observed in the HC group.

#### 3.3.2 ROI12, Right Occipital Thalamus

When the right occipital thalamus (Otha. R, ROI12) was used as the seed ROI, a significant main effect of group was observed for functional connectivity with several frontal and temporal regions after FDR correction (**Supplementary Table 4**). Adjusted by Bonferroni correction, post-hoc analyses showed that FC between Otha. R and these regions were significantly greater in both FES and UHR groups than in HC, with FES patients exhibiting stronger FC than UHR individuals in the bilateral inferior orbitofrontal cortex, left medial superior frontal cortex, and left middle temporal cortex. In addition, the UHR group showed significantly higher FC than did HC in all regions listed above (**Supplementary Table 4**).

### 3.4 Laterality Index of Thalamus Volume

To explore the volume laterality of the thalamus across the three groups, the LI and |LI| were calculated. The results indicated that in all three groups, the thalamus and its nuclei regions were skewed to the right (LI < 0). The |LI| of VM showed a significant group difference (F = 6.176, *p*-FDR = 0.002, and post-hoc analysis showed a significantly lower volume in the FES than in the HC group (*p*-_Bonf_ = 0.002, Table 3).

### 3.5 Correlation Between Neuroimaging Feature and Clinical Symptom/SCFA

All correlation analyses were corrected for multiple comparisons using the false discovery rate (FDR) method.

#### 3.5.1 SCFAs

**Fig. 4.**
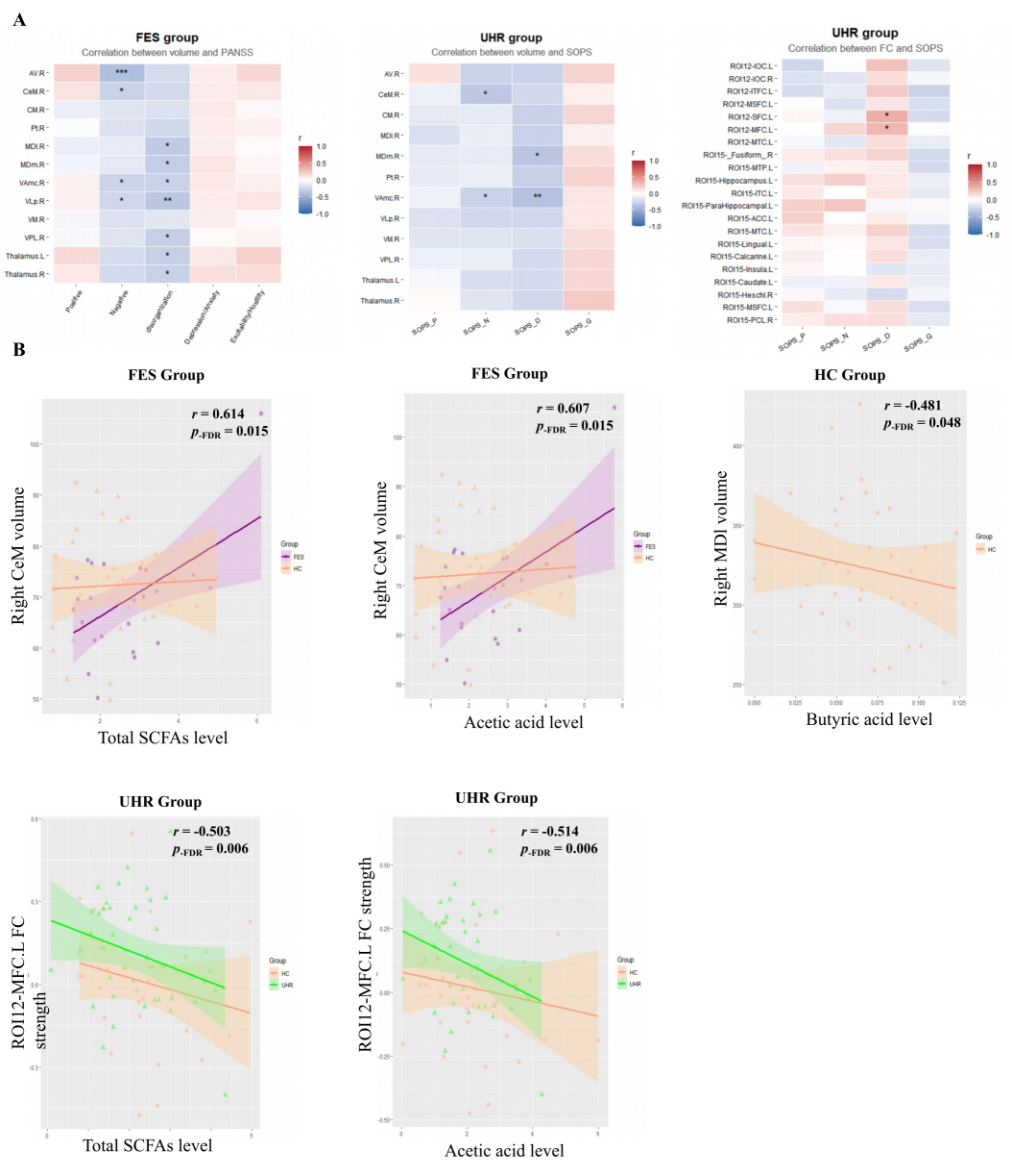
**Correlation between thalamic volume/functional connectivity and clinical symptoms/SCFA levels**. (A) Correlations between thalamic volume/functional connectivity and clinical symptoms. (B) Correlations between thalamic volume/functional connectivity and SCFA levels. * *p*-_FDR_ < 0.05, ** *p*-_FDR_ < 0.01, *** *p*-_FDR_ < 0.001. Note: Sample sizes – volume analysis: FES = 102, UHR = 72, HC = 69; functional connectivity analysis: FES = 76, UHR = 63, HC = 61. Shaded areas around the regression lines represent the 95% confidence interval. Other abbreviations are provided in **Supplementary Table 3**.

As for the thalamic volume, in FES patients, acetic acid (*r* = 0.607, *p*-_FDR_ = 0.015) and the total SCFAs levels (*r* = 0.614, *p*-_FDR_ = 0.015) showed a moderate positive correlation with the volume of the CeM nucleus. In the HC group, butyric acid was moderately negatively correlated with the volume of the MDl nucleus (*r* = –0.481, *p*-_FDR_ = 0.048). No significant volume-SCFA correlations were observed in the UHR group. Regarding functional connectivity, a moderate negative correlation was observed between the strength of Otha. R-left MFC and both acetic acid (*r* = –0.514, *p*-_FDR_ = 0.006) and total SCFAs (*r* = –0.503, *p*-_FDR_ = 0.006) in the UHR group. No significant FC-SCFA correlations were found in the FES or HC groups (Fig. 4).

#### 3.5.2 Clinical Symptoms

Changes in thalamic nuclei volumes were associated with negative and disorganization symptoms in both FES and UHR groups after FDR correction. In FES patients, negative-symptom-score showed mild-to-moderate negative correlations with several right thalamic nucleus (AV, *r* = –0.389, *p*-_FDR_ < 0.001; CeM, *r* = –0.269, *p*-_FDR_ = 0.04; VAmc, *r *= –0.257, *p*-_FDR_ = 0.03; VLp, *r *= –0.243,* p*-_FDR_ = 0.0425), and the disorganization score correlated with bilateral thalamus (left, *r* = –0.294,* p*-_FDR_ = 0.02; right, *r* = –0.277,* p*-_FDR_ = 0.035) and the right thalamic nuclei (MDl, *r* = –0.317,* p*-_FDR_ = 0.01; MDm, *r *= –0.297,* p*-_FDR_ = 0.015; VAmc, *r *= –0.274,* p*-_FDR_ = 0.03; VLp, *r *= –0.338,* p*-_FDR_ = 0.005; VPL, *r* = –0.293,* p*-_FDR_ = 0.02). In the UHR group, the SOPS negative score negatively correlated with the right thalamic nuclei (CeM, *r* = –0.327,* p*-_FDR_ = 0.028; VAmc, *r* = –0.274,* p*-_FDR_ = 0.048), and the disorganization score showed negative correlations with MDm (*r* = –0.35,* p*-_FDR_ = 0.012) and VAmc (*r* = –0.37,* p*-_FDR_ = 0.008). Additionally, we observed greater FC of Otha. R-left SFC (*r* = 0.375,* p*-_FDR_ = 0.012) and left MFC (*r* = 0.327,* p*-_FDR_ = 0.044) were associated with a greater SOPS-D score (Fig. 4, **Supplementary Tables 5.1–5.2**).

## 4. Discussion

The present study has three main findings: (1) A smaller thalamic volume and widespread dysregulation of connectivity were found in the bilateral thalamus. It revealed that alterations in the thalamus are present at the UHR stage, with these changes being more pronounced in the FES group; (2) Thalamic subregions exhibited distinct properties, with smaller volume observed in the right thalamus and its associated nuclei - AV, CeM, CM, MDl, MDm, Pt, VAmc, VLp, VM, VPL in the FES group. Dysfunction was detected in the left sensory thalamus and right occipital thalamus, these alterations were associated with distinct neural circuits in both the UHR and FES group; (3) Severe negative and disorganized symptoms were associated with smaller thalamic nuclei volumes in both FES and UHR groups. In addition, a stronger FC correlated with more severe disorganized symptoms in those two groups; (4) The total SCFAs and acetic acids level were positively correlated with CeM volume, and were negatively correlated with FC of ROI12-left MFC in the UHR group. Butyric acid showed a negative correlation with MDl volume in the HC group. To our knowledge, this is the first study to investigate both drug-naïve FES patients and UHR individuals with clinical assessments; the results suggested that higher SCFA levels may be associated with less thalamic volume reduction in FES patients and altered functional connectivity in UHR subjects. However, these findings should be interpreted cautiously due to the limited number of significant associations and the exploratory nature of the analyses.

We observed a smaller bilateral thalamus volume in FES patients, with a smaller volume in the right thalamus of the FES group than in the UHR group. Subregional differences in thalamic volume were observed on the right side, specifically, the VM nucleus showed significant lateralization in FES patients, suggesting that the lower volume in the right thalamic nucleus was more pronounced than in the left, supporting the possibility that early SCZ has a greater effect on the right thalamus. A large number of studies have reported a smaller bilateral thalamic volume in SCZ and FES patients, with fewer neurons than in HCs [14,17,39,40,41,42,43,44,45], and the right thalamic nuclei were more affected [27]. However, several studies [17,18,46] suggested that thalamic volumes may differ between stage-mixed SCZ and FES patients, with distinct patterns in early and chronic SCZ. These inconsistent results may have been influenced by antipsychotic treatment and disease stage. Our study avoided these confounds and confirmed that the alterations in the right thalamus are more prominent during the early stages of the illness. As the disease progresses, a more pronounced reduction in the left thalamus occurs, and these changes were driven by disease the itself. We also found that the volume reduction of the right thalamus precedes SCZ onset and worsens as the disease progresses, suggesting that it may serve as a potential biomarker for early intervention.

Our subregion analysis found that, FES patients exhibited lower volume in several right thalamic nuclei than did HCs, especially the AV, mediodorsal (MD), and VM. These findings corroborated the work of Hoang et al. [47], who also reported a lower volume of the anterior nuclei in FES patients. The AV nucleus, which is primarily connected to the limbic system, plays a key role in the hippocampal memory circuits, and memory impairment. It has been highlighted as a significant predictor of UHR conversion to psychosis [19], and is a critical subcortical region related to the pathophysiology of SCZ [47]. The MD and VM nuclei are higher-order subregions that engage in reciprocal interactions with the prefrontal cortex [48,49], and the MD nucleus, in particular, is thought to be characteristic of SCZ to some extent [50,51]. A post-mortem study examining neuron counts in the MD and AV nuclei of the thalamus suggested that SCZ is associated with significant neuronal loss in thalamic nuclei that interact with the prefrontal cortex and limbic system [52]. Therefore, we speculated that the lower volume of the AV nucleus was already present at the UHR stage and may have been more closely linked to the risk of conversion to psychosis in UHR individuals. Besides, the volumes of the MDm, VM and VPL nuclei in the FES group were significantly smaller than those of UHR patients. This suggests that changes in these nuclei occur in the early stages of SCZ, potentially serving as an early diagnostic marker. As the disease progresses, more thalamic nuclei are affected, indicating that thalamic abnormalities in UHR are more localized than in FES. Early intervention that targets specific subregions of the thalamus may help to prevent or delay disease onset.

Another noteworthy finding in this study is the dysregulation between the left sensory thalamus and the cortico-striatal-thalamic-cortical (CSTC) circuits, as well as between the right occipital thalamus and the fronto-temporal cortex in both the FES and UHR groups. These neural circuits are modulated by dopamine and are involved in motor control, executive functions, sensory information integration, emotional regulation, attention, and perception [53,54]. Impairment in these circuits has been linked to dysfunctional executive function and persistent symptoms in SCZ, including reality distortion, disorganization, and psychomotor poverty [7]. A previous study has shown that SCZ and FES patients exhibited more widespread and predominantly disrupted FC network damage than did the UHR sample [55]. Notably, our study showed that, compared to HC subjects, FES patients exhibited hyper-connectivity in most brain regions within these circuits, which may have reflected either error or negative correlation. In contrast, UHR individuals demonstrated unstable connectivity abnormalities. It is reasonable to suppose that individuals in the UHR state exhibit widespread FC disruption between the thalamus and cortex. As the disease progresses, those who transition to SCZ may exhibit thalamic hyper-connectivity as a compensatory mechanism for structural abnormalities, potentially contributing to symptom onset. Additionally, it is widely believed that connectivity in SCZ is generally reduced, primarily based on studies involving chronic or medicated patients; enhanced or unstable connectivity is seen in drug-naive and early-stage patients [9]. Longitudinal studies are needed to validate this. Our study extended previous findings by localizing CSTC and fronto-temporal cortex connectivity abnormalities to the sensory and occipital thalamus, thereby providing insights into the potential mechanisms of, and their impact on the disease. In summary, abnormalities in specific thalamic subregions are more localized and sensitive than those in the entire thalamus, potentially serving as early indicators for SCZ or predictive markers for the transition from the UHR state to SCZ.

Consistent with previous studies [56,57,58], our study found that smaller thalamic nuclei volumes were associated with more severe clinical symptoms, supporting the notion that thalamic connections are tightly integrated and that alterations in thalamic nuclei have a broad impact on SCZ patients. The AV nucleus volume showed a strong correlation with negative symptoms in FES patients, suggesting its involvement in negative symptoms early in the disease. The CeM and VAmc nuclei exhibited a stable negative correlation with negative symptoms in both the FES and UHR groups, and similar stable relationships were found between the MDm and VAmc nuclei with disorganization symptoms. Previous evidence was confirmed by the finding that reduced thalamic volume was associated with the severity of disorganized [59] and negative symptoms [60]. These findings may suggest that CeM, VAmc, and MDm nuclei influence symptoms during the UHR state. After disease onset, more thalamic nuclei and the entire thalamus were affected, contributing to symptom severity regulation. Consistent with our findings, Mørch-Johnsen et al. [50] noted that the anterior and medial pulvinar are particularly relevant to negative symptoms. Many studies have also found that the structure and function of the right thalamus and its subregions correlate with negative symptoms, though the specific subregions varied [18,61,62]. That points to the complexity of the relationship between subtle microstructural alterations in a brain region and clinical symptoms.

Our findings also provided limited evidence for the role of short-chain fatty acids in thalamic abnormalities in early SCZ. Although we previously reported altered SCFA levels in FES and UHR groups [32], the current study found no significant group differences in acetic acid levels. Therefore, we could not conclude that reduced acetic acid drives thalamic volume reduction in patients. We observed a potential protective effect of higher acetic acid levels specifically in the CeM nucleus of patients, but this was limited to one of 25 thalamic nuclei. Additionally, the weak negative correlation between butyric acid and MDl volume in healthy controls was absent in both UHR and FES groups. Butyric acid is known for its anti-inflammatory and neuroprotective properties [63], and the disruption of this relationship in patient groups may have indicated impaired gut-brain communication in early SCZ. Although SCFAs may play a modulatory role in neurostructural changes, our results did not support a strong association between SCFAs and thalamic anomalies. Future studies with larger samples are needed to clarify the potential role of gut metabolites in SCZ pathophysiology.

### Limitations

Several limitations should be noted: (1) The relatively small sample size, particularly for the measurement of SCFAs, may have limited statistical power of the study. Future studies with larger samples are needed to adequately address this question. (2) Factors such as diet, BMI, smoking, and inflammatory markers were not available and could not be controlled. (3) Different atlases were used for functional and structural analyses, which limited the depth of discussion. Future studies should use integrated atlases for both structural and functional analyses.

## 5. Conclusions

This multimodal imaging study found that localized, mild abnormalities in the thalamus and its subregions were present prior to the onset of SCZ, suggesting these as potential neurobiological vulnerabilities. The thalamic subregions are intricately connected but showed notable heterogeneity, particularly in the anterior and mediodorsal nuclei. The volume of certain thalamic subregions correlated with negative and disorganized symptoms, with subtle associations detectable even at the UHR stage. Additionally, serum SCFAs may have contributed to SCZ pathogenesis, affecting thalamic functional connectivity during the UHR stage and contributing to volume changes after disease onset. These findings offer valuable insights for early detection and intervention in schizophrenia.

## Data Availability

The raw data supporting the conclusions of this article will be made available by the authors, without undue reservation.
