# Supplementary material for: Thalamic Subregion Alterations and Short-Chain Fatty Acids in Schizophrenia and Ultra-High-Risk Individuals: A Cross-Sectional Study
Source: Alpha Psychiatry. 2026 Jun 29;27(3):49150. doi: 10.31083/AP49150 (PMC13339799; doi:10.31083/AP49150)
Supplement: Supplementary file 1 [file 2757-8038-27-3-49150-s1.zip › Supplementary Materials.docx]

**Supplementary Table 1** Interaction Effects of Group with Covariates Using GLM (Wilks' Lambda)

|  | F | *p* | ***η*2** |
| --- | --- | --- | --- |
| group * gender | 1.071 | 0.351 | 0.116 |
| group * age | 0.892 | 0.682 | 0.099 |
| group * years of education | 0.788 | 0.85 | 0.088 |
| group * eTIV | 1.284 | 0.102 | 0.136 |

Note: The table presents the Wilks' Lambda statistic, along with the corresponding F, p-value, and partial eta-squared (η²) for each interaction term. The model assessed the interaction between group (FES, UHR, HC) and covariates (gender, age, years of education, eTIV) using General Linear Models (GLM). The threshold for statistical significance was set at *p* < 0.05.

Supplementary Table 2.1 Demographic and clinical characteristic of included subjects in functional MRI analysis

|  | **FES**  **(n=76)** | **UHR**  **(n=63)** | **HC**  **(n=61)** | **F/χ²** | ***p-*value** |
| --- | --- | --- | --- | --- | --- |
| Age | 22.03±6.49 | 18.97±4.64 | 21.69±4.82 | 7.28 | <.001^a***^ |
| Gender(male/female) | 45/31 | 33/30 | 33/28 | 0.72 | 0.698^b^ |
| The years of education | 12.13±2.71 | 11.21±2.90 | 13.41±2.53 | 10.52 | <.001^a***^ |
| *PANSS 5 factor model* |  | | | | |
| Positive symptom | 21.51±7.13 | - | - | - | - |
| Negative symptom | 21.32±5.60 | - | - | - | - |
| disorganization | 26.29±6.94 | - | - | - | - |
| Depression/Anxiety | 18.74±5.47 | - | - | - | - |
| Excitability/Hostility | 21.99±6.05 | - | - | - | - |
| *SOPS* |  | | | | |
| SOPS-P score | - | 10.78±4.93 | - | - | - |
| SOPS-N score | - | 12.11±5.16 | - | - | - |
| SOPS-D score | - | 5.08±2.67 | - | - | - |
| SOPS-G score | - | 4.63±2.94 | - | - | - |

Note: SOPS, scale of prodromal symptoms; P, positive symptom; N, negative symptom; D, disorganized symptom; G, general symptom. The data are described as (Mean ± SD). *p* < 0 .05 was considered statistically significant. ***: *p* < 0.01. a: The ***p-*value** was obtained by a one-way analysis of variance (ANOVA).

b: The ***p-*value** were obtained by chi-square test.

Supplementary Table 2.2 Demographic characteristic of included subjects in SCFAs analysis.

|  | **FES** | **UHR** | **HC** | **F/*χ*²** | *p-value* |
| --- | --- | --- | --- | --- | --- |
| Gender (male/female) | 33/26 | 25/26 | 24/16 | 1.15 | 0.562 |
| Age (year) | 20.92 ± 5.59 | 19.00 ± 4.75 | 19.25 ± 2.62 | 2.34 | 0.102 |
| Years of education | 11.10 ± 2.85 | 11.00 ± 2.55 | 12.92 ± 2.56 | 7.19 | <0.001*** |

Note: The data are described as (Mean ± SD). One-way ANOVA was used for the age, years of education. The Chi-square test was used for gender comparison. *p* < 0 .05 was considered statistically significant. ***: *p* < 0.001.

Supplementary Table 2.3 Comparisons of Demographics and Clinical Characteristics between sMRI, fMRI, and SCFA groups.

|  | **FES** | | | | | **UHR** | | | | | **HC** | | | | |
| --- | --- | --- | --- | --- | --- | --- | --- | --- | --- | --- | --- | --- | --- | --- | --- |
|  | **Structural (n=102)** | **Functional**  **(n=76)** | **SCFA**  **(n=59)** | **χ²/F** | ***p-*value** | **Structural (n=72)** | **Functional (n=63)** | **SCFA**  **(n=51)** | **χ²/F** | ***p-*value** | **Structural (n=69)** | **Functional (n=61)** | **SCFA**  **(n=40)** | **χ²/t** | ***p-*value** |
| Age | 21.23±5.54 | 22.03±5.49 | 20.92±5.59 | 0.761 | 0.468 | 19.00±4.72 | 18.97±4.64 | 19.00±4.75 | 0.001 | 0.999 | 21.78±4.62 | 21.69±4.82 | 19.25±2.62 | 5.085 | 0.007** |
| Sexr (male/female) | 59/43 | 45/31 | 33/26 | 0.147 | 0.930 | 37/33 | 33/30 | 25/26 | 0.233 | 0.890 | 39/30 | 33/28 | 24/16 | 0.241 | 0.886 |
| The years of education | 11.75±2.84 | 12.13±2.71 | 11.10±2.85 | 2.263 | 0.106 | 11.06±2.78 | 11.21±2.80 | 11.00±2.55 | 0.101 | 0.904 | 13.58±2.53 | 13.41±2.53 | 12.92±2.56 | 1.280 | 0.281 |
| Positive symptom | 21.88±6.85 | 21.51±7.13 | 21.83±7.77 | 0.063 | 0.939 | - | - |  | - | - | - | - |  | - | - |
| Negative symptom | 21.48±6.32 | 21.32±5.60 | 21.61±8.24 | 0.032 | 0.968 | - | - |  | - | - | - | - |  | - | - |
| disorganization | 27.08±7.19 | 26.29±6.94 | 26.88±7.32 | 0.275 | 0.760 | - | - |  | - | - | - | - |  | - | - |
| Depression/Anxiety | 18.92±5.36 | 18.74±5.47 | 18.86±6.02 | 0.023 | 0.977 | - | - |  | - | - | - | - |  | - | - |
| Excitability/Hostility | 22.10±5.51 | 21.99±6.05 | 22.03±5.25 | 0.009 | 0.991 | - | - |  | - | - | - | - |  | - | - |
| SOPS-P score | - | - |  | - | - | 10.78±4.93 | 10.68±5.05 | 11.80±5.64 | 0.750 | 0.474 | - | - |  | - | - |
| SOPS-N score | - | - |  | - | - | 12.11±5.16 | 12.43±5.21 | 11.54±6.51 | 0.428 | 0.652 | - | - |  | - | - |
| SOPS-D score | - | - |  | - | - | 5.08±2.67 | 5.14±2.68 | 4.02±2.41 | 3.009 | 0.052 | - | - |  | - | - |
| SOPS-G score | - | - |  | - | - | 4.63±2.94 | 4.67±2.87 | 4.76±3.01 | 0.032 | 0.968 | - | - |  | - | - |

Note: Clinical symptoms were assessed using the Scale of Prodromal Symptoms (SOPS), including positive (P), negative (N), disorganized (D), and general (G) symptom domains. Continuous variables are presented as mean ± standard deviation. Group differences were evaluated using one-way ANOVA for continuous variables and chi-square tests for categorical variables ( gender). **: *p* < 0.01.

Supplementary Table 3. Abbreviation

| Abbreviation | Full term | Abbreviation | Full term |
| --- | --- | --- | --- |
| FES | Drug-naive first-episode schizophrenia | MSFC | medial superior frontal cortex |
| UHR | Ultra-high risk | PCL | paracentral Lobule |
| HC | Healthy control | IOC | inferior orbitofrontal cortex |
| PANSS | Positive and Negative Syndrome Scale | ITFC | inferior triangle frontal cortex |
| SOPS | Scale of Prodromal Symptoms | SFC | superior frontal cortex |
| P | Positive symptom score | MFC | medial frontal cortex |
| N | Negative symptom score | AV | Anteroventral |
| D | Disorganized symptom score | CeM | Central medial |
| G | General symptom score | CM | Centromedian |
| SCFA | Short chain fatty acid | MDl | Mediodorsal lateral parvocellular |
| eTIV | estimated total intracranial volume | MDm | Mediodorsal medial magnocellular |
| Stha.L(ROI5) | Left sensory thalamus | Pt | Paratenial |
| Otha.R (ROI12) | Right occipital thalamus | VAmc | Ventral anterior magnocellular |
| MTP | middle temporal pole | VLp | Ventral lateral posterio |
| ITC | inferior temporal cortex | VM | Ventromedial |
| ACC | anterior cingulate cortex | VPL | Ventral posterolateral |
| MTC | middle temporal cortex | ROI | Region of Interest |

**Supplementary Table 4** Significant Functional Connectivity Changes in Thalamic Subregions.

| **Seed** | **Brain regions** | **Cluster size** | **Peak MNI coordinates** | | | **Peak T value** | **Linear Model**  **(FES vs UHR vs HC)** | | | **Post-hoc**  **(**Bonferroni-adjusted) | **95% CI** |
| --- | --- | --- | --- | --- | --- | --- | --- | --- | --- | --- | --- |
|  |  |  | x | y | z |  | ***F*** | ***p-_FDR_***  **(FDR correction)** | ***η*2** |  | **[Lower,Upper]** |
| **ROI5 left sensory thalamus** | right fusiform | 21 | 42 | -12 | -36 | 9.39 | 11.314 | <0.001*** | 0.103 | FES>UHR, *p_-Bonf_* < 0.001;  FES>HC, *p_-Bonf_* < 0.001 | [0.07,0.18]  [0.04,0.16] |
|  | left middle temporal pole (MTP.L) | 60 | -36 | 18 | -36 | 12.22 | 13.239 | <0.001*** | 0.118 | FES>UHR, *p_-Bonf_* < 0.001;  FES>HC, *p_-Bonf_* = 0.012;  HC>UHR, *p_-Bonf_* = 0.015 | [0.09,0.21]  [0.02,0.13]  [0.01,0.13] |
|  | left hippocampus | 39 | -30 | -6 | -27 | 10.34 | 12.692 | <0.001*** | 0.114 | FES>UHR, *p_-Bonf_* < 0.001;  FES>HC, *p_-Bonf_* = 0.023;  HC>UHR, *p_-Bonf_* = 0.010 | [0.09,0.20]  [0.009,0.12]  [0.02,0.13] |
|  | left inferior temporal cortex (ITC.L) | 31 | -45 | -33 | -24 | 10.52 | 12.340 | <0.001*** | 0.111 | FES>UHR, *p_-Bonf_* < 0.001;  FES>HC, *p_-Bonf_* = 0.016;  HC>UHR, *p_-Bonf_* = 0.018 | [0.08,0.18]  [0.01,0.12]  [0.01,0.12] |
|  | left parahippocampal | 42 | -18 | -18 | -21 | 11.04 | 14.729 | <0.001*** | 0.130 | FES>UHR, *p_-Bonf_* < 0.001;  HC>UHR, *p_-Bonf_* < 0.001 | [0.07,0.17]  [0.06,0.17] |
|  | left anterior cingulate cortex (ACC.L) | 24 | -9 | 24 | -3 | 14.91 | 11.273 | <0.001*** | 0.103 | FES>UHR, *p_-Bonf_* = 0.042;  HC>FES, *p_-Bonf_* = 0.004;  HC>UHR, *p_-Bonf_* < 0.001 | [0.002,0.11]  [0.03,0.14]  [0.08,0.20] |
|  | left middle temporal cortex (MTC.L) | 186 | -42 | -63 | 9 | 11.76 | 12.243 | <0.001*** | 0.111 | FES>UHR, *p_-Bonf_* < 0.001  FES>HC, *p_-Bonf_* < 0.001 | [0.09,0.23]  [0.05,0.19] |
|  | Left Lingual | 40 | -6 | -63 | -3 | 7.92 | 9.487 | <0.001*** | 0.088 | FES>UHR, *p_-Bonf_* < 0.001;  FES>HC, *p_-Bonf_* = 0.033;  HC>UHR, *p_-Bonf_* = 0.039 | [0.10,0.28]  [0.007,0.181]  [0.005,0.186] |
|  | Left Calcarine | 120 | 0 | -93 | 12 | 16.74 | 16.028 | <0.001*** | 0.140 | FES>UHR, *p_-Bonf_* < 0.001;  HC>UHR, *p_-Bonf_* < 0.001 | [0.10,0.23]  [0.09,0.23] |
|  | Left Insula | 34 | -33 | -6 | 18 | 9.81 | 14.633 | <0.001*** | 0.129 | FES>UHR, *p_-Bonf_* < 0.001;  HC>UHR, *p_-Bonf_* < 0.001 | [0.05,0.17]  [0.11,0.23] |
|  | Left Caudate | 22 | -9 | 3 | 15 | 11.99 | 14.584 | <0.001*** | 0.129 | UHR>FES, *p_-Bonf_* < 0.001;  UHR>HC, *p_-Bonf_* = 0011  HC>FES, *p_-Bonf_* = 0.008 | [0.10,0.22]  [0.02,0.14]  [0.02,0.14] |
|  | Right HeschI | 22 | 33 | -27 | 15 | 9.60 | 10.165 | <0.001*** | 0.094 | HC>UHR, *p_-Bonf_* < 0.001;  HC>FES, *p_-Bonf_* = 0.021;  FES>UHR, *p_-Bonf_* = 0.017 | [0.09,0.24]  [0.01,0.15]  [0.01,0.15] |
|  | left medial superior frontal cortex (MSFC.L) | 29 | -9 | 57 | 21 | 9.31 | 9.295 | <0.001*** | 0.086 | FES>UHR, *p_-Bonf_* < 0.001;  FES>HC, *p_-Bonf_* < 0.001 | [0.06,0.19]  [0.06,0.19] |
|  | Right Paracentral Lobule (PCL.R) | 460 | 9 | -30 | 69 | 10.59 | 14.078 | <0.001*** | 0.125 | FES>UHR, *p_-Bonf_* < 0.001;  HC>UHR, *p_-Bonf_* < 0.001 | [0.10,0.23]  [0.06,0.20] |
| **ROI12 right occiptal thalamus** | left inferior orbitofrontal cortex (IOC.L) | 91 | 48 | 30 | -15 | 14.63 | 12.739 | <0.001*** | 0.115 | FES>HC, *p_-Bonf_* < 0.001;  FES>UHR, *p_-Bonf_* = 0.035；  UHR>HC, *p_-Bonf_* = 0.005 | [0.10,0.22]  [0.005,0.129]  [0.03,0.16] |
|  | right inferior orbitofrontal cortex (IOC.R) | 25 | -3 | 57 | -18 | 12.26 | 14.155 | <0.001*** | 0.126 | FES>HC, *p_-Bonf_* < 0.001  FES>UHR, *p_-Bonf_* = 0.017  UHR>HC, *p_-Bonf_* = 0.005 | [0.11,0.24]  [0.01,0.14]  [0.03,0.17] |
|  | left inferior triangle frontal cortex (ITFC.L) | 91 | -48 | 33 | -9 | 12.68 | 12.762 | <0.001*** | 0.115 | FES>HC, *p_-Bonf_* < 0.001  UHR>HC, *p_-Bonf_* < 0.001 | [0.09,0.22]  [0.05,0.18] |
|  | left medial superior frontal cortex (MSFC.L) | 69 | -57 | -54 | 3 | 16.32 | 12.134 | <0.001*** | 0.110 | FES>UHR, *p_-Bonf_* = 0.01;  FES>HC, *p_-Bonf_* < 0.001;  UHR>HC, *p_-Bonf_* = 0.028 | [0.02,0.15]  [0.09,0.22]  [0.008,0.141] |
|  | Left superior frontal cortex (SFC.L) | 62 | -9 | 57 | 18 | 11.82 | 12.674 | <0.001*** | 0.114 | FES>HC, *p_-Bonf_* < 0.001;  UHR>HC, *p_-Bonf_* < 0.001 | [0.07,0.20]  [0.08,0.21] |
|  | left medial frontal cortex (MFC.L) | 89 | -51 | 15 | 24 | 12.66 | 10.156 | <0.001*** | 0.093 | FES>HC, *p_-Bonf_* < 0.001  UHR>HC, *p_-Bonf_* = 0.013 | [0.08,0.21]  [0.02,0.15] |
|  | left middle temporal cortex (MTC.L) | 31 | 0 | 39 | 48 | 14.11 | 15.100 | <0.001*** | 0.133 | FES>UHR, *p_-Bonf_* = 0.004;  FES>HC, *p_-Bonf_* < 0.001;  UHR>HC, *p_-Bonf_* = 0.014 | [0.03,0.15]  [0.11,0.24]  [0.02,0.15] |

Note: the voxel wise threshold was set at *p_-_*_FDR_ < 0.05 after FDR correction, the cluster size was set at 20. x, y, z: coordinates of peak locations in the Montreal Neurological Institute (MNI) space; ***: *p_-_*_FDR_ < 0.001. Post-hoc pairwise were adjusted by Bonferroni correction: *P_-Bonf_* < 0.05 considered significant. Other abbreviations are listed in Supplementary Table 1.

**Supplementary Table 5.1** Partial Correlation Between Volume and Clinical Indices

|  |  | Left thalamus | Right thalamus | Right AV | Right CeM | Right CM | Right MDl | Right MDm | Right Pt | Right VAmc | Right VLp | Right VM | Right VPL |
| --- | --- | --- | --- | --- | --- | --- | --- | --- | --- | --- | --- | --- | --- |
| *PANSS score* | | | | | | | | | | | | | |
| Positive symptom | *r* | 0.172 | 0.097 | 0.213 | 0.13 | -0.1 | -0.101 | -0.057 | -0.011 | 0.065 | 0.056 | -0.049 | -0.038 |
|  | *p_-FDR_* | 0.119 | 0.3489 | 0.073 | 0.344 | 0.449 | 0.546 | 0.731 | 0.920 | 0.534 | 0.588 | 0.798 | 0.815 |
| Negative symptom | *r* | -0.206 | -0.225 | -0.389 | -0.269 | -0.154 | -0.119 | -0.131 | -0.175 | -0.257 | -0.243 | -0.173 | -0.164 |
|  | *p_-FDR_* | 0.077 | 0.073 | 0.0005*** | 0.04* | 0.340 | 0.546 | 0.380 | 0.223 | 0.03* | 0.0425* | 0.403 | 0.283 |
| Disorganization | *r* | -0.294 | -0.277 | -0.207 | -0.22 | -0.17 | -0.317 | -0.297 | -0.235 | -0.274 | -0.338 | -0.145 | -0.293 |
|  | *p_-FDR_* | 0.02* | 0.035* | 0.073 | 0.080 | 0.340 | 0.01* | 0.015* | 0.110 | 0.03* | 0.005** | 0.403 | 0.02* |
| Depression / Anxiety | *r* | 0.113 | 0.139 | 0.093 | 0.085 | 0.095 | 0.081 | 0.125 | 0.085 | 0.095 | 0.087 | 0.076 | 0.024 |
|  | *p_-FDR_* | 0.274 | 0.226 | 0.369 | 0.412 | 0.449 | 0.546 | 0.380 | 0.648 | 0.534 | 0.505 | 0.770 | 0.815 |
| Excitability / Hostility | *r* | 0.206 | 0.152 | 0.18 | 0.113 | 0.033 | 0.017 | 0.035 | 0.067 | 0.075 | 0.124 | 0.023 | 0.046 |
|  | *p_-FDR_* | 0.077 | 0.226 | 0.101 | 0.344 | 0.749 | 0.867 | 0.735 | 0.648 | 0.534 | 0.385 | 0.825 | 0.815 |
| *SOPS* | | | | | | | | | | | | | |
| SOPS-P | *r* | -0.045 | 0.017 | 0.145 | -0.091 | -0.133 | -0.194 | -0.129 | -0.116 | -0.078 | -0.173 | -0.147 | -0.091 |
|  | *p_-FDR_* | 0.460 | 0.890 | 0.2370 | 0.541 | 0.287 | 0.226 | 0.311 | 0.420 | 0.529 | 0.211 | 0.231 | 0.460 |
| SOPS-N | *r* | -0.2 | -0.147 | -0.269 | -0.327 | -0.131 | -0.108 | -0.125 | -0.099 | -0.274 | -0.24 | -0.154 | -0.103 |
|  | *p_-FDR_* | 0.460 | 0.311 | 0.054 | 0.028* | 0.287 | 0.509 | 0.311 | 0.420 | 0.048* | 0.098 | 0.231 | 0.460 |
| SOPS-D | *r* | -0.224 | -0.227 | -0.273 | -0.23 | -0.279 | -0.282 | -0.35 | -0.286 | -0.37 | -0.245 | -0.185 | -0.224 |
|  | *p_-FDR_* | 0.264 | 0.126 | 0.054 | 0.118 | 0.084 | 0.080 | 0.012* | 0.072 | 0.008** | 0.098 | 0.231 | 0.264 |
| SOPS-G | *r* | 0.181 | 0.242 | 0.203 | 0.075 | 0.191 | 0.073 | 0.15 | 0.199 | 0.109 | 0.109 | 0.149 | 0.179 |
|  | *p_-FDR_* | 0.286 | 0.126 | 0.128 | 0.541 | 0.238 | 0.5540 | 0.311 | 0.208 | 0.504 | 0.3780 | 0.2310 | 0.286 |

Note: The threshold of statistical significance was set at *p_-_*_FDR_ < 0.05 under the FDR correction. *: *p_-_*_FDR_ < 0.05, **: *p_-_*_FDR_ < 0.01, ***: *p_-_*_FDR_ < 0.001. Other abbreviations are listed in Supplementary Table 1.

**Supplementary Table 5.2** Partial Correlation Between Functional Connectivity and SOPS Scores in the UHR Group

|  |  | SOPS-P | SOPS-N | SOPS-D | SOPS-G |
| --- | --- | --- | --- | --- | --- |
| ROI12 - left IOC | *r* | -0.24 | 0.012 | 0.281 | -0.156 |
|  | *p_-FDR_* | 0.130 | 0.930 | 0.116 | 0.311 |
| ROI12 - right IOC | *r* | -0.173 | -0.151 | 0.151 | -0.046 |
|  | *p_-FDR_* | 0.332 | 0.332 | 0.332 | 0.726 |
| ROI12 - left ITFC | *r* | -0.207 | -0.125 | 0.236 | -0.266 |
|  | *p_-FDR_* | 0.151 | 0.341 | 0.138 | 0.138 |
| ROI12 - left MSFC | *r* | -0.006 | -0.168 | 0.109 | -0.192 |
|  | *p_-FDR_* | 0.963 | 0.398 | 0.543 | 0.398 |
| ROI12 - left SFC | *r* | 0.042 | -0.084 | 0.375 | -0.219 |
|  | *p_-FDR_* | 0.750 | 0.695 | 0.012* | 0.186 |
| ROI12 - left MFC | *r* | 0.002 | 0.192 | 0.327 | 0.008 |
|  | *p_-FDR_* | 0.987 | 0.286 | 0.044* | 0.987 |
| ROI12 - left MTC | *r* | -0.045 | -0.135 | 0.15 | -0.11 |
|  | *p_-FDR_* | 0.735 | 0.536 | 0.536 | 0.536 |
| ROI5 - right fusiform | *r* | 0.091 | 0.139 | 0.198 | -0.224 |
|  | *p_-FDR_* | 0.491 | 0.387 | 0.258 | 0.258 |
| ROI5 - left MTP | *r* | -0.073 | 0.061 | 0.078 | -0.225 |
|  | *p_-FDR_* | 0.645 | 0.645 | 0.645 | 0.336 |
| ROI5 - left hippocampus | *r* | 0.14 | 0.221 | 0.105 | -0.095 |
|  | *p_-FDR_* | 0.468 | 0.360 | 0.468 | 0.468 |
| ROI5 - left ITC | *r* | 0.097 | -0.005 | 0.109 | -0.127 |
|  | *p_-FDR_* | 0.468 | 0.360 | 0.468 | 0.468 |
| ROI5 - left parahippocampal | *r* | 0.222 | 0.265 | -0.045 | 0.027 |
|  | *p_-FDR_* | 0.178 | 0.164 | 0.837 | 0.837 |
| ROI5 - left ACC | *r* | 0.224 | 0.015 | 0.093 | -0.053 |
|  | *p_-FDR_* | 0.344 | 0.907 | 0.907 | 0.907 |
| ROI5 - left MTC | *r* | 0.126 | 0.055 | 0.214 | -0.21 |
|  | *p_-FDR_* | 0.451 | 0.677 | 0.214 | 0.214 |
| ROI5 - left Lingual | *r* | 0.044 | 0.019 | 0.107 | -0.203 |
|  | *p_-FDR_* | 0.884 | 0.884 | 0.830 | 0.476 |
| ROI5 - left Calcarine | *r* | 0.079 | -0.001 | 0.154 | -0.114 |
|  | *p_-FDR_* | 0.733 | 0.996 | 0.733 | 0.733 |
| ROI5 - left Insula | *r* | 0.045 | 0 | -0.139 | -0.116 |
|  | *p_-FDR_* | 0.977 | 0.999 | 0.760 | 0.760 |
| ROI5 - left Caudate | *r* | -0.088 | -0.072 | 0.099 | -0.111 |
|  | *p_-FDR_* | 0.584 | 0.584 | 0.584 | 0.584 |
| ROI5 - right HeschI | *r* | 0.035 | -0.122 | -0.208 | -0.062 |
|  | *p_-FDR_* | 0.788 | 0.710 | 0.444 | 0.788 |
| ROI5 - left MSFC | *r* | 0.144 | -0.029 | 0.148 | -0.187 |
|  | *p_-FDR_* | 0.364 | 0.826 | 0.364 | 0.364 |
| ROI5 - right PCL | *r* | 0.076 | 0.147 | 0.149 | -0.131 |
|  | *p_-FDR_* | 0.562 | 0.425 | 0.425 | 0.425 |

Note: The threshold of statistical significance was set at *p_-_*_FDR_ < 0.05 under the FDR correction. Abbreviations are listed in Supplementary Table 1.
